# Selfish, promiscuous and sometimes useful: how mobile genetic elements drive horizontal gene transfer in microbial populations

**DOI:** 10.1098/rstb.2021.0234

**Published:** 2022-10-10

**Authors:** Matthieu Haudiquet, Jorge Moura de Sousa, Marie Touchon, Eduardo P. C. Rocha

**Affiliations:** Institut Pasteur, Université de Paris Cité, CNRS UMR3525, Microbial Evolutionary Genomics, Paris 75015, France

**Keywords:** horizontal gene transfer, evolution, defence systems, bacteriophages, satellites, plasmids

## Abstract

Horizontal gene transfer (HGT) drives microbial adaptation but is often under the control of mobile genetic elements (MGEs) whose interests are not necessarily aligned with those of their hosts. In general, transfer is costly to the donor cell while potentially beneficial to the recipients. The diversity and plasticity of cell–MGEs interactions, and those among MGEs, result in complex evolutionary processes where the source, or even the existence of selection for maintaining a function in the genome, is often unclear. For example, MGE-driven HGT depends on cell envelope structures and defense systems, but many of these are transferred by MGEs themselves. MGEs can spur periods of intense gene transfer by increasing their own rates of horizontal transmission upon communicating, eavesdropping, or sensing the environment and the host physiology. This may result in high-frequency transfer of host genes unrelated to the MGE. Here, we review how MGEs drive HGT and how their transfer mechanisms, selective pressures and genomic traits affect gene flow, and therefore adaptation, in microbial populations. The encoding of many adaptive niche-defining microbial traits in MGEs means that intragenomic conflicts and alliances between cells and their MGEs are key to microbial functional diversification.

This article is part of a discussion meeting issue ‘Genomic population structures of microbial pathogens’.

## 1. Introduction

The gene repertoires of microbial species change very fast and their pangenomes are often orders of magnitude larger than the average genome [1,2]. Most such genes are acquired by horizontal gene transfer (HGT) driven by mobile genetic elements (MGEs). Yet MGEs are autonomous genetic agents that may proliferate even when they have a negative impact on host fitness. Gene flow is thus a rich provider of novel functions to microbial genomes but is largely out of the control of the recipient cells. On the one hand, this means that microbial adaptation depends heavily on the trade-off between gaining advantageous functions by MGE-driven HGT and the costs associated with these elements. On the other hand, as genomes contain many MGEs and these often interact antagonistically, gene flow is shaped by a complex interplay between the host and its many MGEs, as well as between the MGEs themselves. These interactions depend on the characteristics of the MGEs and on the host genetic background, notably its ability to control infections of deleterious MGEs and to integrate the novel genetic information. Ultimately, many rare genes in microbial populations may be effectively under selection because they are adaptive for the MGEs carrying them. Whether this affects cell fitness, and in which sense, it is most often unclear. Here, we review how MGEs drive, but also constrain microbial evolution by HGT. While our text focuses on bacteria, where mechanisms are better known and examples more abundant, it is often also applicable to the interactions between Archaea and their MGEs. We start by a short summary of the mechanisms of transfer of MGEs, highlighting recent findings on their interactions.

## 2. Genomes as playgrounds of mobile genetic elements

**Figure 1.**
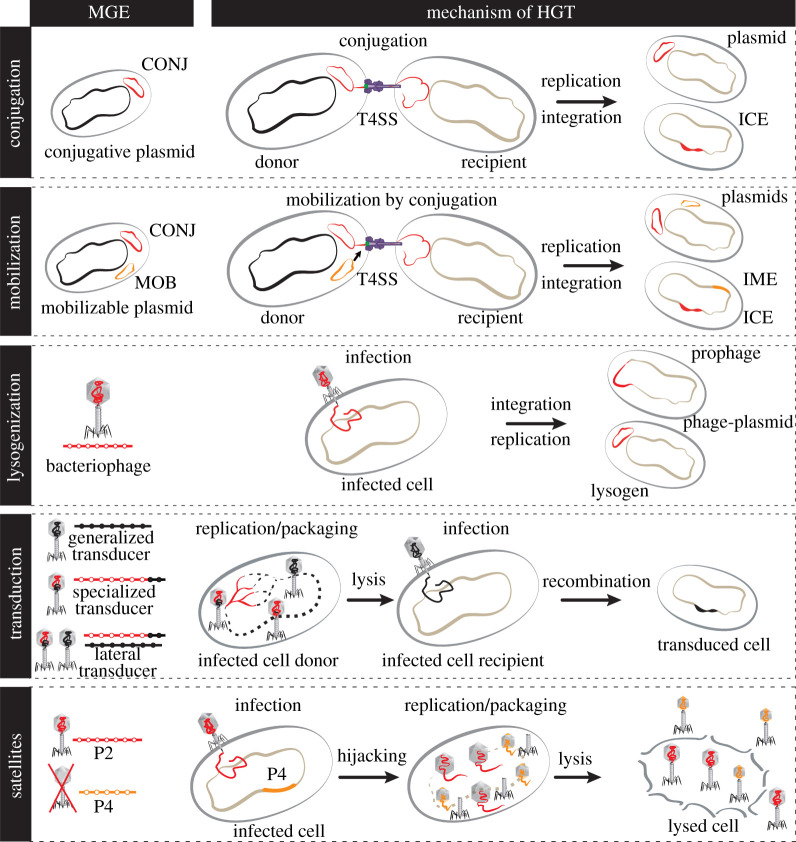
Major mechanisms of HGT driven by MGEs. CONJ, conjugative element; MOB, element mobilizable by conjugation. T4SS, type IV secretion system; ICE, integrative conjugative element; IME, integrative mobilizable element.

MGEs drive DNA transfer between bacteria either by transferring themselves between cells or by mediating the transfer of chromosomal DNA (figure 1). Some mechanisms of HGT do not depend on MGEs [3], most notably natural transformation [4], but their relevance across bacteria in the acquisition of novel genes remains to be understood (e.g. [5]). In this review, we focus on the role of MGEs as drivers of HGT and will not expand on these other processes. MGEs can be classified in terms of their mechanisms of autonomous horizontal (conjugation or viral particles) or vertical transmission (extrachromosomal or integrative). There is extensive genetic diversity within each type of MGE, which can complicate their identification and characterization. Furthermore, some MGEs are parasites or competitors of other MGEs, establishing complex ecological dynamics within populations.

The most frequent mechanism of conjugation involves a relaxase that nicks and attaches to a single strand of DNA. The nucleoprotein filament is then transferred between physically close cells by a type IV secretion system, resulting in the replication of the element [6]. Conjugation can transfer vast amounts of DNA, up to entire chromosomes. Conjugative elements are called plasmids when extrachromosomal, and integrative conjugative elements (ICEs) when they integrate the chromosome. Despite a clear distinction made between these two types of elements in the literature, they encode similar conjugative machineries for horizontal transmission and are present across most bacterial clades [7]. Moreover, ICEs capable of autonomous replication and plasmids integrated in chromosomes have been described [8,9], suggesting the existence of few differences between the two types of elements.

The ability of conjugative elements to transfer between cells can be exploited by mobilizable elements that are present in the same host. Interactions between mobilizable and conjugative elements have been studied more in detail in plasmids. Mobilizable plasmids are typically smaller than conjugative plasmids and do not encode the conjugative pilus required for autonomous HGT. Some encode a relaxase that interacts with pili encoded by conjugative elements present in the cell. Such mobilizable plasmids are at least as abundant as conjugative plasmids and tend to encode similar types of traits [10]. Many other plasmids lack even a relaxase and their mechanisms of transfer, as well as their interactions with other MGEs, are poorly understood. Despite the exploitative interaction between these two types of MGEs, it is not known whether this systematically imparts a significant cost for the conjugative plasmid.

The contribution of temperate bacteriophages (phages) for HGT is complicated by their role as bacterial predators. Upon cell entry, temperate phages can opt between active reproduction and cell lysis (lytic cycle), or lysogeny, where they replicate synchronously with the host either integrated in the chromosome or as phage-plasmids. Half of the available bacterial genomes are recognizably lysogens [11], and some prophages encode traits adaptive to the host, like virulence factors and bacteriocins [12], but can also kill their hosts by induction of the lytic cycle [13]. The effect of temperate phages in bacterial fitness may thus depend on physiological and environmental conditions (see below). Phages can also transfer bacterial genes by generalized, specialized or lateral transduction [14,15]. Each mechanism differentially impacts the scope and efficiency of transfer of bacterial traits. For example, specialized transduction transfers only a few chromosomal genes in the neighbourhood of the prophage, whereas generalized transduction transfers genes from across the chromosome. Lateral transduction occurs when phage replication starts while the prophage is still integrated in the chromosome, and can result in the transfer of extensive neighbouring chromosomal regions [16]. Of note, the amount of DNA packaged by phages is limited by the virion size, which in temperate phages tends to accommodate around 50 kb (with large variations across phages). As a result, a bacterial chromosome can only be transferred by transduction when fragmented across multiple virions. But since cells can liberate many phages, the extent of bacterial DNA transferred by transduction can be huge. A back-of-the-envelope calculation has estimated that a single lysate of phages that infect *Staphylococcus aureus* has the potential to encode up to 20 000 copies of an entire bacterial chromosome in transduction particles [17].

Despite being parasites of bacteria, phages have their own parasites. Phage satellites are small mobile elements (*ca* 7–18 kb) lacking components of the viral particle for autonomous transfer. Instead, they encode sophisticated mechanisms to hijack the particles of ‘helper’ phages to transfer between cells [18]. Three main types of phage satellites have been described: P4 in Enterobacterales [19], phage-inducible chromosomal islands in Enterobacterales and Firmicutes [20], and phage-inducible chromosomal island-like elements (PLEs) in *Vibrio* spp. [21]. Many other types of satellites may still be uncovered, and those that are known seem very abundant and diverse. For example, almost half of *Escherichia coli* genomes have between one and three P4-like satellites [19]. Phage satellites can impact their bacterial hosts at different levels: by transducing chromosomal DNA [15], by encoding virulence factors [22], or by encoding anti-MGE defense systems [23]. Satellites are costly to phages because they hijack their particles, thereby decreasing phage burst size. However, there is significant variation in this cost, depending on the satellite-helper pair. Some PLEs completely abolish phage reproduction [24], whereas P4 has, under certain conditions, a much lower impact on phage reproductive fitness [25].

**Figure 2.**
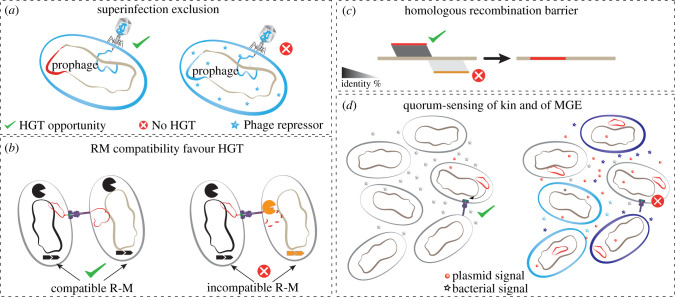
Recombination, defense and communication shape HGT. (*a*) Prophages protect against other phages by many mechanisms, including superinfection exclusion and repression of gene expression. (*b*) Plasmids can eavesdrop the quorum-sensing mechanisms of the host cell and use their own to promote their conjugation when there are many closely related hosts without plasmids in the neighbourhood of the host cell. (*c*) Homologous recombination requires high similarity between the exogenous DNA and the chromosome. (*d*) Bacteria with compatible restriction-modification (R-M) systems can exchange DNA at higher rates because the DNA is marked with the correct epigenetic markers and is not restricted by the recipient cell.

As satellites are mobilized by phages and mobilizable plasmids by conjugative elements, there are other MGEs that can be mobilized by these parasites of parasites [26,27]. This makes them parasites of parasites of parasites of bacteria (which may themselves be parasites of Eukaryotes). While the full scope of ecological interactions between all these MGEs is not very well known, it is clearly a multi-layered complex network that opens paths for both conflicts and alliances in the cell. As an example of such complex interactions, prophages interact not only with other prophages and satellites, e.g. by repressing or actively targeting them [28] (figure 2*a*), but also with other MGEs, particularly with conjugative or mobilizable elements, which can encode anti-phage defenses [29] or be mobilized by phages [30]. Further, and despite their potential costs for bacterial reproduction, there are also synergies between MGEs and host cells: phage satellites encode defense systems against phages that they cannot parasitize, which favours the other MGEs in the genome, including prophages, and the host cell [23]. Finally, MGEs can exchange genetic material between them, and with their host, through transposable elements [31] or different recombination mechanisms [32]. For example, a chromosomal gene conferring resistance to carbapenem antibiotics in *Pseudomonas aeruginosa* originated from a conjugative plasmid, with the transfer from plasmid to bacterial chromosome likely being mediated by transposases [33].

The abundance and diversity of MGEs, and the myriad of their possible interactions, establish a scenario where bacteria are a playground for MGEs and their genomes are shaped by the associated eco-evolutionary conflicts. The following sections of this review will thus address the different ways in which these interactions affect the networks of gene transfer that shape microbial evolution.

## 3. Interplay between ecology and mobile genetic elements shapes horizontal gene transfer

The transfer of a MGE requires either that cells meet for conjugation, or that viral particles diffuse far enough to find susceptible hosts. Therefore, the size and the diversity of the gene pool for a species depend on the composition of microbial communities. Metagenomics data have shown that transfers occur more frequently between isolates from similar environments [34,35]. Similar conclusions were obtained by searching for highly similar genes across different genomes [36,37]. These results have spurred proposals that the dynamic interplay among hosts, MGEs and environments shapes networks of genetic exchanges within communities [38]. Accordingly, the lineages that are most prevalent across different habitats within *Listeria* spp. have higher rates of HGT [39]. The frequency of genetic exchanges mediated by MGEs is expected to depend on the density of cell hosts in the community, which may explain why the densely populated human gut is a hotspot of genetic exchanges [34,40]. It also depends on the physical distances that can be covered by MGEs outside of the cell. These distances are extremely small for conjugative elements because they require direct cell–cell contact for transfer. Phages can survive for long periods of time in the environment [41], which allows their dispersion across large geographical distances, e.g. in aquatic environments. Hence, phage-driven HGT is more likely to result in direct transfers across segregated microbial communities than conjugation.

Structured environments, like biofilms, are thought to be the most frequent types of microbial environments on the planet [42]. The structure of the environment is important because it shapes the physiological response of individual cells, the networks of interactions between microbes and the transmission dynamics of their MGEs [43]. Conjugative systems mate more efficiently on solid surfaces [44,45] and conjugation can thus take place at very high rates on the outer layers of biofilms [46,47]. Plasmids that lack adaptive genes for their hosts and are only maintained through high transfer frequencies are thus more likely to persist on biofilms [48]. Interestingly, conjugation itself spurs the formation of biofilms [49], thus driving conditions that effectively favour the transfer of conjugative elements. In contrast, limited diffusion of phage particles hinders phage amplification in structured environments, thereby decreasing the generation of phage genetic diversity and making phage–host antagonistic coevolution less predictable [50–52]. Habitat structure and composition are therefore key determinants of the rate and type of MGE-driven HGT.

## 4. Mobile genetic element manipulation of the timing of gene transfer

Several mechanisms increase the rates of genetic exchanges under conditions of maladaptation, i.e. when the acquisition of novel functions is more likely to have a positive impact on fitness. Expression of competence for natural transformation is usually under the control of conserved regulatory circuits of the recipient cell, even if several plasmids have been described to repress transformation [53,54]. In most other cases, the decision for transfer is under the control of MGEs, not of the host or recipient cells. In theory, investments in horizontal and vertical transmission are equally important for the success of the MGE at the evolutionary time scale [55]. Hence, very costly MGEs are expected to have lower rates of vertical transmission but can still prosper if their rates of HGT are high. The investment in the different types of transmission may vary. When the host's viability is at risk, the investment in horizontal transmission is much more rewarding that the investment in vertical transmission. This results in an intense exodus of MGEs from the cell to increase their chances of survival, corresponding to a shift in investment from vertical to horizontal transmission in the search for better hosts. The consequence for microbial populations is an increase in the rates of HGT.

MGEs can sense cues that indicate the cell is no longer a promising host for vertical transmission, and thus shift their investment from vertical towards horizontal transmission. For example, certain DNA lesions lead to the activation of the SOS response, which favours the induction of prophages [56,57] and conjugative elements [58]. Because of their effect on cell physiology, including induction of SOS in some bacteria, antibiotics can spur the transfer of phages [59] and conjugative plasmids [60]. Inflammatory responses in the gut also increase conjugative transfer and prophage induction, fostering the spread of functions such as those associated with virulence and antibiotic resistance [61,62]. These processes are under the control of the MGEs and can be costly, and sometimes lethal, to the donor cells. Occasionally, they result in the acquisition of adaptive genes by a recipient cell.

The timing and source of gene flow in populations may also be conditioned by social processes. Quorum-sensing allows bacteria to assess the abundance of closely related cells in a population. Similarly, MGEs have evolved to sense bacterial quorum-sensing signals to eavesdrop on bacterial communication and decide when to invest in horizontal transmission [63]. MGEs also encode their own quorum-sensing systems that further inform them about the presence of similar elements in neighbouring bacteria. Conjugative plasmids use it to transfer between cells when the environment is crowded with closely related bacteria that lack the plasmid [64,65] (figure 2*d*). Temperate phages use it to favour lysogeny when the density of similar phages in the environment is high [66] and to induce the lytic cycle when the concentration of susceptible hosts is high [67,68]. Although systems of molecular communication have only recently been uncovered in MGEs, it is possible that several other strategies of communication underlie their interactions with other MGEs and with their potential hosts [69].

## 5. Scope of horizontal gene transfer as the result of mobile genetic element–host interactions

Since much of HGT relies on the ability of MGEs to transfer horizontally between hosts, their host range will determine the rate at which adaptive traits can be transferred across different species. In general terms, the efficiency of HGT decreases with the phylogenetic distance between donor and recipient cells [70]. The magnitude of this effect depends on the mechanism of transfer of MGEs. Conjugative elements, which do not require specific cell receptors, often have large host ranges and can transfer elements across genera or even phyla [71]. Phage host ranges are usually narrower and can be limited to a small number of strains having a specific cell receptor or serotype [72] (see below). The host range of the many MGEs that exploit other MGEs to transfer across cells is poorly known. Some mobilizable plasmids might have a very broad host range because they can hijack conjugative systems from different conjugative plasmids [27]. Similarly, the host range of phage satellites depends on their ability to hijack multiple phages.

Once an MGE has successfully passed the envelope and the cell defense barriers, it still endures functional constraints because the molecular mechanisms used by the MGE for horizontal transmission (e.g. production of viral particles or conjugative pili) may not work in the novel genetic background, thereby restricting the MGE's effective host range. For example, conjugative pili are specialized to specific membrane structures and those functioning in cells with an outer membrane usually do not work in cells lacking it [73]. How functions related to vertical transmission work (or do not work) in the novel genetic background of recipient cells also contributes to explaining differences in host range. Site-specific recombinases allow MGEs to integrate at highly conserved regions of the chromosome, like tRNA genes, without inactivating them [74]. These integrases function in very different genetic backgrounds, facilitating transfer of MGEs across distantly related taxa with little fitness impact for the host. The higher sensitivity of plasmid replicases to the genetic background relative to ICE integrases contributes to explaining why the latter have even broader host ranges than the former [75]. The broad host range of conjugative elements and their high genetic plasticity may explain why these elements are the major vectors of the ongoing large-scale transfer of antibiotic resistance from soil bacteria to human pathogens [76].

DNA integrating into the genome by homologous recombination must have high sequence identity with the chromosome (figure 2*c*) [77]. This mechanism is important for allelic exchanges in core genes, which in many species result in rates of introduction of nucleotide changes higher than those caused by mutation rates [78]. In bacteria that are not naturally transformable, these allelic exchanges require MGE-driven HGT. Yet core genes are systematically absent from MGEs. Conjugation or transduction are the most likely candidates to provide the chromosomal DNA required for allelic exchanges. Recent studies show that lateral transduction can drive the transfer of vast amounts of chromosomal DNA within species [79]. However, we still lack quantitative measures of the relative importance of these different processes in shaping patterns of recombination in natural populations. While recombination might allow the integration of exogenous DNA, it may also favour the deletion of MGEs from the chromosome [80]. Unfortunately, most of these recombination processes leave very few, if any, traces of the vehicle of transfer of the exogenous DNA into the cell, which is also why the real-world impact of some types of HGT are still so difficult to quantify (e.g. generalized transduction). As a result, the mechanisms of acquisition of exogenous DNA allowing allelic exchanges in core genes by homologous recombination remain largely hypothetical and based on extrapolation from data of laboratory experiments.

## 6. Mobile genetic elements–cell envelope interactions are key to successful transfer

MGE-driven HGT requires an initial interaction between the recipient cell envelope and the structural component of the MGE that interfaces with it, be it the tip of the conjugative pilus or the tail of the phage. Viral particles interact with cells via phage-encoded receptor-binding proteins (RBPs), which enable their adsorption and stabilization at the cell surface before DNA is injected into the cell [81]. RBPs are very specific to their corresponding bacterial receptors and shape the host range of the phage and the sensitivity of the bacterium. By contrast, conjugation is much less reliant on a specific receptor at the cell envelope [82]. These mechanistic differences contribute to explaining why phages tend to have narrower host ranges than conjugative elements.

**Figure 3.**
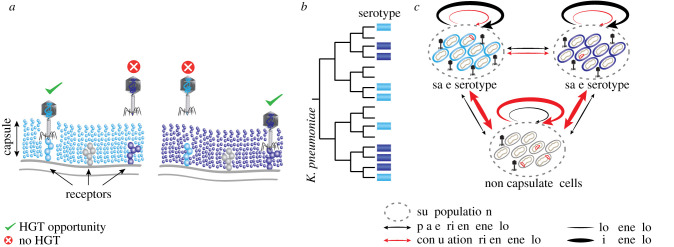
(*a*) The capsule is a barrier to phage infection when it hides phage receptors. But some phages have evolved to degrade the capsule and can thus use it for adsorption. (*b*) The capsule is frequently lost and gained by HGT during *K. pneumoniae* evolution, resulting in frequent serotype switching. (*c*) Because of the capsular specificity of temperate phages in *K. pneumoniae*, phage-driven HGT is much more frequent within than between serotypes. By contrast, non-capsulated cells are more permissive to conjugation. Hence, gene flow depends on the presence of the capsule, its serotype and the type of MGE driving HGT.

Structures located at the cell envelope, like the bacterial capsule, provide additional control over the access of MGEs to the cell. Capsules are composed of membrane-bound polysaccharide chains and constitute the first point of contact of MGEs with the cell [83]. They can be very large, creating exclusion zones thicker than the cell diameter, and protect bacteria from agents like macrophages or antimicrobial peptides [84]. They can also protect from phages, because capsules can hide phage receptors [85]. Capsules were thus thought to decrease gene flow [86]. However, phages that infect bacteria that constitutively express their capsule, like *Klebsiella pneumoniae* and *Acinetobacter baumannii*, have evolved to use the capsule to adsorb to the cell [87]. The RBPs of these types of phages are endowed with capsule depolymerases, specific to one or a few capsular serotypes, granting them access to the outer membrane after adsorption at the capsule (figure 3*a*). But this adaptation comes at a cost: such phages may become dependent on a specific capsule to adsorb efficiently to the cell envelope, and are unable to infect non-capsulated cells, or even cells with a different capsular serotype. This is not a rare occurrence since the temperate phage infection networks of *K. pneumoniae* show clear serotype-specific clusters [88], resulting in more frequent phage-driven gene flow between strains with similar serotypes [89] (figure 3*b*). The requirement for a capsule for phage adsorption implies that phage pressure may lead to selection for capsule inactivation, because non-capsulated bacteria are resistant to these phages [88]. Interestingly, such non-capsulated cells are not sexually isolated because even if phage-driven transfer may be diminished, they are much more receptive to conjugative elements [89]. Hence, variations in the capsule composition or expression change both phage and conjugation-driven gene flow. The consequences of these changes are very different and somewhat complementary (figure 3*c*): phage-driven transfer is particularly high between strains of the same serotype and conjugation is more frequent towards non-capsulated strains.

Many other components of the cell envelope are involved in complex interplays with MGEs and affect their rates of transfer. The O-antigen of lipopolysaccharide (LPS) is often targeted by phages, and it displays high genetic and chemical variability within and across species [90]. Switching from smooth to rough LPS type is usually associated with phage resistance and altered LPS structures. Since LPS-related rough phenotypes are also associated with modified virulence in pathogens [91], phage predation also impacts the evolution of virulence in these strains. The dependence of MGE transfer on the physiological traits of cells means that changes in envelope composition can reshape networks of gene flow and this will eventually also affect the HGT of components of the envelope. In conclusion, bacterial physiology, and the different selective pressures impacting it, are a strong determinant of both the frequency and type of MGE-driven HGT.

## 7. Cell and mobile genetic element defenses and counter-defenses constrain gene flow

Once the DNA enters the recipient cell cytoplasm, defense systems can still block its expression. Microbes and their MGEs have evolved numerous specialized defense and counter-defense systems that are frequently gained and lost. Their genetic diversification is caused by the antagonistic coevolution between microbial cells and MGEs. These defense systems are currently being uncovered at a fast pace and have recently been reviewed [92–94]. Interestingly, recent data suggest that most such ‘cellular’ defense systems are actually encoded in MGEs and not in conserved sections of the host chromosome [95]. The available evidence is thus that MGE-encoded defense systems are protecting their host cell as a side-effect of their action to protect the MGE from other MGEs [96]. Antagonistic coevolution between MGEs could thus be at least as important as that between MGEs and the host.

One might think that there is a trade-off between maintaining many defense systems and allowing the genome to acquire adaptive genes by HGT. Since defense systems block some MGEs from certain lineages, they carve preferential pathways of gene flow in microbial populations. Notably, there is more HGT and homologous (allelic) recombination between pairs of strains with compatible restriction modification (R-M) systems, by far the most abundant specialized defense systems, than between other strains. This is because MGEs transferred between strains with compatible R-M systems carry the same methylation patterns and thus are able to escape restriction that would otherwise prevent their DNA from establishing in the cell [97] (figure 2*b*). While defense systems tend to limit the income of new DNA, in certain circumstances they may even facilitate HGT [98]. Many defense systems, like viperins or retrons [99,100], target very specific functions and may not impact the transfer of most MGEs. Hence, defense systems shape but do not abolish gene flow in microbial populations. MGEs, being both targets and producers of defense systems, are both vectors of and barriers to HGT.

## 8. Mobile genetic elements turnover

**Figure 4.**
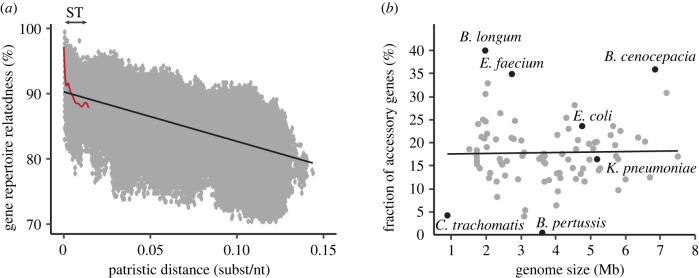
Impact of the high turn-over of MGEs on gene repertoire (left) and genome size of the host. (*a*) Gene repertoire relatedness decreases quickly with the patristic distance in *E. coli* (red spline fit line) at short evolutionary distances, i.e. between genomes of the same sequence types (ST). The subsequent changes are more moderated and approximately linear with time (black linear fit line) [103]. Of note, the variance around these average trends is very large. This figure was simplified and redrawn from the data in [103]. (*b*) The horizontal line is the linear regression of the fraction of accessory genes per genome as a function of the average species genome size (for the 90 most-represented species in GenBank). Figure redrawn and simplified from the results presented in [105]. *B. cenocepacia,* *Burkholderia cenocepacia*; *B. longum*, *Bifidobacterium longum*; *C. trachomatis*, *Chlamydia trachomatis*; *E. coli*, *Escherichia coli*; *E. faecium*, *Enterococcus faecium*; *K. pneumoniae*, *Klebsiella pneumoniae*; *B. pertussis*, *Bordetella pertussis*.

MGEs represent a large fraction of the accessory genome of many species, but they are rarely maintained in a lineage for a long period of time [95,101]. These rapid dynamics of gene gain and loss contribute to the U-shaped distribution of the frequency of gene families in pangenomes, typically resulting in a large majority of gene families being either very frequent (persistent genome) or quite rare (usually acquired in MGEs) [102]. The high turnover of MGEs means that closely related strains can have very different MGE contents. This is the case in *E. coli* and *K. pneumoniae*, where epidemiologically indistinguishable strains (from the same sequence types) differ in the many different MGEs they carry [103,104]. A high MGE turnover also means that while MGEs are a sizeable part of bacterial genomes (*ca* 10% in *E. coli* for phages plus plasmids) they account for most of its variation in size [103]. This rapid flux of MGEs explains why relatedness between gene repertoires decreases very quickly with phylogenetic distance for closely related genomes (figure 4*a*).

Many forces drive the rapid turnover of MGEs and their genes in bacterial genomes [37,106]. Foremost, MGEs can be very costly and their hosts counter-selected [107]. Induction of temperate phages kills the host, and even plasmids and transposons may involve lower, but not necessarily negligible, costs [108,109]. The rapid loss of MGEs could thus be interpreted as the result of their negative contribution to the host fitness. In this view, the ubiquitous presence of MGEs in microbial populations could be explained by their selfish spread.

However, extensive data suggest a more nuanced view of the costs and benefits of HGT driven by MGEs [110]. The costs of MGEs can decrease rapidly after their acquisition by a host, as frequently observed in plasmids. The acquisition of novel plasmids is usually associated with an elevated physiological burden, but purifying selection does not necessarily lead to plasmid loss or chromosomal integration of beneficial genes [106], especially when the element carries adaptive traits under positive selection [111]. In such cases, there is rapid emergence of compensatory mutations, either in the chromosome or in the plasmid themselves, that alleviate the cost of the element [112], e.g. by resolving specific genetic conflicts [113]. Amelioration contributes to lower the cost of MGEs as parasites and increases their stability in microbial lineages.

Many MGEs carry genes that are adaptive under specific and potentially transient conditions [2]. The linkage between these adaptive genes and the MGE may provide the ensemble with positive net fitness advantage to the host for some time. The MGE would be selectively maintained as long as these genes provide a sufficient fitness advantage, but could be quickly lost when its positive impact on fitness ceases. Many accessory genes in MGEs may be adaptive for only short periods. For example, antibiotic resistance genes tend to be costly and are typically lost when individuals are no longer subject to antibiotics [114]. Genes under negative frequency-dependent selection, e.g. toxins encoded by MGEs associated with inter or intra-specific competition [115], are also expected to be rapidly replaced. The presence of genes adaptive only in particular contexts means that the associated MGEs may endure fluctuating types of selection, i.e. they are adaptive in certain contexts and parasites in others.

Finally, neutral processes may accelerate the loss of MGEs. Adaptive genes may escape costly MGEs by translocating into the chromosome [116], thereby turning an adaptive MGE into a costly one that becomes counter-selected even if the host fitness has not changed. MGEs may also be affected by the pervasive bias toward deletions in bacteria [117] that may be more pronounced in MGEs because they have many transposable elements [118] and repeated DNA [119]. Therefore, the high turnover of MGEs is probably the result of multiple selective pressures and mutational biases that operate at different scales: the gene, the MGE and the host genome.

## 9. Impact of mobile genetic element turnover on pangenome evolution

The rapid turnover of MGEs implies that high rates of HGT do not necessarily result in larger microbial genomes. Except for very small genomes that sometimes show little or no evidence of MGEs and HGT, there is extensive variation in the frequency of accessory genes per microbial genome. This frequency varies from a few percent to close to 40% [105], with many species showing values between 10 and 25% (figure 4*b*). Species with large genomes tend to have higher effective population sizes [120], but they do not necessarily have very high rates of HGT [121], nor of homologous recombination [120]. The fraction of the genome that corresponds to the accessory genome is also not correlated with the average species genome size [105]. Hence, the fraction of accessory genes, most of which are acquired by HGT, does not seem to result from the same selection processes that result in larger genomes. Instead, it may reflect the rates and costs of gene gain and loss. Since most HGT seems to be driven by MGEs, the persistence of novel genes in bacterial lineages will be dependent on deletion biases, on the fitness effect of the gene and on its direct genetic environment (the MGE). If the MGEs have high horizontal transmission rates, they are also more likely to be costly. Hence, genomes with high rates of HGT might only have an average amount of accessory genes because most acquired genes are in costly MGEs that are rapidly lost from the genome (or the genome is purged from populations by purifying selection).

Extreme reductions in genome size have been observed in endo-mutualists that are sexually isolated, endure population bottlenecks, and live in constant environments [122]. But similar processes of genome reduction have been found in free-living bacteria that are able to exchange DNA, presumably due to selection for genome streamlining [123]. Surprisingly, bacterial genomes can shrink despite being under the influence of high rates of HGT. The phylogroups of *E. coli* with the smallest genomes have the highest rates of gene repertoire diversification and fewer but more diverse MGEs [103]. Many of these small *E. coli* genomes are from freshwater isolates, lack antibiotic resistance genes and virulence factors, and have a large pangenome. They seem to be locally adapted to their nutrient-poor environment. This example illustrates how ecological opportunities can shape the number, the type and the distribution of MGEs in a population. In this case, while high gene flow may have facilitated parallel adaptation to an environment that is very different from the mammalian gut, selection for streamlining in such nutrient-poor environments [123] has likely resulted in genome reduction.

## 10. Outlook and unsolved mysteries

The identification of the pertinent levels of selection—genes, MGEs or/and genomes—can be extremely complicated when populations have many MGEs that are prone to genetic conflicts. Because a lot of HGT is driven by MGEs, many of the most recent genes in the genome may be neutral or deleterious to the host cell, while being selected due to the benefits they confer to the MGE itself. Still, genes in MGEs can sometimes be adaptive to the host as a by-product of their selection by the MGE, typically because higher host fitness increases the fitness of the MGE encoding the trait. This is the case for many traits in plasmids and phages, like antibiotic resistance, toxins and defense systems that are adaptive both to the MGE and to its host. Many such genes may be adaptive under certain situations and not in others. For example, phage satellites can block phage infections and thus favour the bacterial host, but may be costly when the specific helper phages are absent. Likewise, prophages without genes that are adaptive to the host might still provide resistance to other similar phages. While the qualitative understanding of these processes has much progressed, there is a paucity of quantitative data to understand how much of the HGT is potentially of adaptive value for the recipient cell.

MGEs can be costly and reproduce selfishly across populations but may also occasionally provide adaptive genomic changes by increasing genome evolvability [124]. Many studies revealed the roles of transposable elements in shuttling adaptive genes between replicons, thereby favouring their transfer in plasmids or their stabilization in the chromosome [118]. But transposition of these elements also results in frequent pseudogenization of useful genes. How frequently the gains in evolvability provided by MGEs compensate the costs of these elements is poorly known. These indirect selective effects (i.e. higher-order selection) are hard to measure in the laboratory because they depend on the genetic diversity of communities and the frequencies and types of ecological challenges faced by Bacteria and Archaea. Further work will be needed to disentangle how and when such elements contribute, or not, to host adaptation. Such studies should account for the fact that recipient cells have little control over the rates of HGT and that MGEs have their own evolutionary interests, meaning that it is difficult to interpret changes in the rates of HGT in the light of selection for microbial evolvability.

The availability of low-cost sequencing and the current focus on the worrisome spread of antibiotic resistance genes by MGEs may provide crucial data to quantify how rates of HGT depend on the type of MGE and its mechanisms of horizontal transmission. For example, phages encode many toxins, but few antibiotic resistance genes [125]. The latter are much more frequent in conjugative elements, especially in plasmids [75]. The genetic plasticity, range of interactions and mode of transfer of MGEs might explain why certain MGEs are preferentially associated with certain traits.

Finally, it is important to stress that many MGEs might still be unknown and many of the known ones have as yet unknown mechanisms of transfer. For example, over 50% of known plasmids do not encode either a conjugative apparatus or a known relaxase [10]. They may be transferred by one of many processes: conjugation using a relaxase from another plasmid [126], generalized transduction [30,127], natural transformation [128] or vesicles [129]. The current lack of information on the mechanisms of transfer of many MGEs raises questions about their origins, mechanisms of dissemination and impact on microbial evolution. Rough estimates suggest that most large contiguous stretches of non-homologous sequences integrated in genomes by integrases, presumably MGEs, remain to be characterized [130]. The identification of these elements and their interactions with hosts and other MGEs will certainly contribute to a better understanding of gene flow in microbial populations.

## Data Availability

This article has no additional data.
